# Prevalence of Reported Mental Illness in Addis Ababa, Ethiopia: A Community-Based Study

**DOI:** 10.4314/ejhs.v34i2.10S

**Published:** 2024-12

**Authors:** Sitota Tsegaye, Hanna Melesse, Hana Sime, Elsabeth Wujira, Semira Abdelmenan, Nebiyou Fasil, Hanna Yemane Berhane, Dongqing Wang, Wafaie Fawzi, Alemayehu Worku, Meaza Demissie, Yemane Berhane

**Affiliations:** 1 Department of Nutrition and Behavioral Science, Addis Continental Institute of Public Health, Addis Ababa, Ethiopia; 2 Department of Epidemiology and Biostatistics, Addis Continental Institute of Public Health, Addis Ababa, Ethiopia; 3 Department of Global Health and Health Policy, Addis Continental Institute of Public Health, Addis Ababa, Ethiopia; 4 Department of Reproductive Health and Population, Addis Continental Institute of Public Health, Addis Ababa, Ethiopia; 5 Department of Global and Community Health, College of Public Health, George Mason University, Fairfax, Virginia, United States of America; 6 Department of Global Health and Population, Harvard T.H. Chan School of Public Health, Harvard University, Boston, Massachusetts, United States of America

**Keywords:** Reported Mental Illness, Addis HDSS, Ethiopia

## Abstract

**Background:**

Mental illness is the second leading cause of disease burden globally, following cardiovascular diseases. Over 25% of people worldwide will experience mental illness at some point, and low-income countries contribute 12% of the global disease burden. However, data on mental disorders are scarce in low-income countries, leading to insufficient attention to mental illness prevention and treatment. This study assesses the prevalence of mental illness and its associated factors in Addis Ababa.

**Methods:**

This study was conducted in the Addis Health and Demography Surveillance System (Addis HDSS) in Yeka sub-city, Addis Ababa, using a structured questionnaire. The prevalence of reported mental illness was calculated with 95% confidence intervals (CIs), and logistic regression was employed to examine factors such as age, sex, chronic illness, physical disability, and wealth index associated with mental illness.

**Results:**

A total of 107,494 respondents participated, with 44.6% men. The average age was 29.23 ± 18.97 years. The reported prevalence of mental illness was 1.1% (95% CI: 1.1–1.2), with 50.7% of those affected being men. Mental illness was more common in individuals aged 65 years and older. The odds of reported mental illness were higher in men (AOR 1.24; 95% CI 1.12–1.39), in those with physical disabilities (AOR 10.12; 95% CI 8.64–11.84), and in those with chronic illness (AOR 2.35; 95% CI 1.22–4.54).

**Conclusion:**

This study adds to the limited data on mental illness prevalence in the community, highlighting it as a significant health burden. The results emphasize the need for healthcare planning focused on mental health and increased community awareness. Particular attention should be given to vulnerable groups, including the elderly, those with physical disabilities, and those with chronic illnesses.

## Introduction

Globally, mental illness accounts for up to 13% of disability-adjusted life years (DALYs), making it the second leading cause of disease burden after cardiovascular diseases(1). Over 25% of people worldwide will experience mental illness at some point in their lives. Low-income countries contribute 12% of the global disease burden(2). In Ethiopia, mental illness is one of the prevalent non-communicable illness, a ccounting for 11% of the overall burden of disease(3). Mental illness, often characterized by depression, mild anxiety, and medically unexplained physical symptoms, affects a significant portion of any community(4). Most mental illnesses begin between the ages of 12 and 24, although symptoms are frequently noticed later in life(5).

Several factors are associated with mental illness, including genetic predisposition, living alone, chronic conditions (e.g., kidney failure)(6), lower educational levels(2,7), physical disability(8), and low socioeconomic status(9–11).

Much of the literature on mental illness focuses on its global burden, prevalence, and the associated socioeconomic costs (12). While mental illnesses can be debilitating and life-threatening, they are often underrecognized and undertreated, particularly in low-income countries. The rise of mental illness is especially notable in urban areas(13). In Ethiopia, mental health issues are frequently not perceived as life-threatening, and thus they receive insufficient attention from policymakers and healthcare providers(14,15). This gap in the literature particularly regarding self-reported mental health motivates the present study. Although many studies have utilized clinical assessments and diagnostic tools, there is a lack of comprehensive research on individuals' subjective experiences and perceptions of their mental health. Self-reported data offers valuable insights into individuals' lived experiences and is crucial for effective mental health intervention. This study aims to address this gap by assessing self-reported mental health in Addis Ababa, contributing to a better understanding of community perceptions and informing targeted mental health interventions.

## Methods

**Study setting**: The study was conducted in the Addis Health and Demography Surveillance System (Addis HDSS), located in the Yeka sub-city of Addis Ababa, Ethiopia. The Addis HDSS site encompasses six woredas and includes 240 enumeration areas. Data were collected from December 2022 to January 2023.

**Study design and population**: This study utilized baseline census data from the Addis HDSS.

**Data collection**: Data were gathered using a structured questionnaire adapted from the Ethiopian Demographic and Health Survey (EDHS). The questionnaire included questions on the presence of mental illness/symptoms for each individual in the household. It was prepared in English and translated into Amharic. Data collection was carried out using electronic devices with Open Data Kit (ODK) software. All household members were considered in the census, but interviews were conducted with individuals present during data collection. Respondents reported the presence of mental illness, physical disabilities, and chronic conditions (such as hypertension, diabetes, and asthma) for each household member.

**Data analysis**: Data analysis was performed using SPSS version 20. The prevalence of reported mental illness was calculated with 95% confidence intervals. The distribution of mental illness across different demographic groups (age, sex, marital status, education, employment, chronic illness, physical disability, and wealth index) was analyzed. The wealth index was constructed using Principal Component Analysis (PCA), incorporating variables such as housing type, ownership, number of bedrooms, water source, sanitation facilities, car ownership, and savings habits.

Logistic regression was used to identify factors associated with mental illness. Variables with a p-value ≤ 0.2 were considered for multivariable analysis. A p-value < 0.05 was considered statistically significant.

**Ethical considerations**: Ethical approval was obtained from the Addis Continental Institute of Public Health and the Addis Ababa Health Bureau. Written informed consent was obtained from all respondents, and confidentiality was maintained by excluding personal identifiers.

## Results

A total of 107,494 respondents participated in the study, with 44.6% male and 55.4% female. The average age of participants was 29.23 ± 18.97 years, with 33.2% aged 20–34. In terms of education, 27.7% had completed primary school, and 20.3% had attended college or higher education. Additionally, 33.5% of participants were married, 43.6% were employed, 8.6% had a chronic illness, and 2.1% had a physical disability. (Table 1)

**Table 1:** Sociodemographic and health-related characteristics of the respondents in Addis HDSS site, 2023 (n = 107,494)

Characteristic		Number (%)
Age (years)	0-4	10,404 (9.7%)
	5-9	8,233 (7.7%)
	10-19	15,579 (14.5%)
	20-34	35,679 (33.2%)
	35-64	31,434 (29.2%)
	65+	6,164 (5.7%)
Sex	Male	47987 (44.6%)
	Female	59507 (55.4%)
Marital status	Never married	35121 (32.7%)
	Currently Married	36052 (33.5%)
	Formerly married	10918 (10.2%)
	Underage	25403 (23.6%)
Education	Preschool	4413 (4.1%)
	Primary	29818 (27.7%)
	Secondary	29189 (27.2%)
	Vocational /technical	4617 (4.3%)
	College/ university	21769 (20.3%)
	Underage	17688 (16.5%)
Employment	Student	17739 (16.5%)
	Unemployed	20243 (18.8%)
	Employed	46885 (43.6%)
	Retired	3990 (3.7%)
	Underage	18637 (17.3%)
Chronic illness	No	9216 (8.6%)
	Yes	98278 (91.4%)
Wealth status	Lowest	19940 (18.5%)
	Second	18042 (16.8%)
	Middle	20588 (19.2%)
	Fourth	23575 (21.9%)
	Highest	25349 (23.6%)
Self-reported physical disability	No	105270 (97.9%)

	Yes	2224 (2.1%)

**Prevalence and associated factors of reported mental illness**: The prevalence of reported mental illness was 1.1% (95% CI: 1.1–1.2), equating to 1,209 individuals out of 107,494. Of these, 50.7% were male. Mental illness was more prevalent among those aged 65 and older (2.7%), followed by individuals aged 35–64 (1.5%). Among those with physical disabilities, 10.7% reported mental illness, and 3.4% of individuals with chronic illness experienced mental health symptoms. The lowest wealth quantile reported the highest rate of mental illness (1.3%).

On the logistic regression model men were 1.24 times more likely to report mental illness than women (AOR 1.24; 95% CI 1.12–1.39). Mental illness was more common among individuals with physical disabilities (AOR 10.12; 95% CI 8.64–11.84) and those with chronic illnesses (AOR 2.35; 95% CI 1.22–4.54). (Table 2)

**Table 2:** Determinants of reported mental illness in Addis HDSS site, 2023

Characteristics		Reported mental illness		Crude OR (95% CI)	Adjusted OR (95% CI)
		
		Yes N (%)	No N (%)		
Age	0-4	75 (0.7%)	10329 (99.3%)	ref	ref
	5-9	53 (0.6%)	8180 (99.4%)	0.89(0.63-1.27)	0.92 (0.65-1.31)
	10-19	120 (0.8%)	15459 (99.2%)	1.07(0.80-1.43)	1.13 (0.84-1.50)
	20-34	312 (0.9%)	35367 (99.1%)	1.22(0.94-1.57)	1.29 (0.99-1.66)
	35-64	480 (1.5%)	30955 (98.5%)	2.14(1.67-2.73)	1.95 (1.52-2.49)
	65+	169 (2.7%)	5995 (97.3%)	3.88(2.95-5.12)	2.56 (1.93-3.39)
Sex	Female	596 (1%)	58911 (99%)	ref	ref
	Male	613 (1.3%)	47347 (98.7%)	1.28(1.14-1.43)	1.24(1.12-1.39)
Chronic	Yes	315 (3.4%)	8901(96.6%)	3.86(3.38-4.39)	2.35(1.22-4.54)
Illness	No	894 (0.9%)	97384 (99.1)	Ref	ref
Physical	Yes	238 (10.7%)	1986 (89.3%)	12.87(11.10-14.93)	10.12(8.64-11.84)
Disability	No	971 (0.9%)	104299 (99.1%)	Ref	ref
Wealth	Lowest	257(1.3%)	19683(98.7%)	1.19(1.00-1.41)	1.19 (0.99-1.41)
index	Second	207(1.1%)	17835(98.9%)	1.06(0.88-1.27)	1.04 (0.86-1.25)
	Middle	206(1.0%)	20382(99.0%)	0.92(0.77-1.11)	0.93 (0.78-1.12)
	Fourth	264(1.1%)	23311(98.9%)	1.03(0.87-1.22)	1.02 (0.86-1.21)
	Highest	275(1.1%)	25074(98.9%)	Ref	ref

## Discussion

The study found a 1.1% prevalence of reported mental illness, with older age, male sex, physical disability, and chronic illness identified as key risk factors. While the prevalence of mental illness in Ethiopia is generally reported to range between 14–32% (4,16), this study's lower prevalence may reflect the use of self-reported data rather than validated diagnostic tools, which likely underestimates the actual burden. In addition, stigma and poor mental health awareness in Ethiopia may contribute to underreporting (17–19).

The higher prevalence of mental illness among those aged 65 and older aligns with findings from other studies(20), suggesting that older adults face increased life stressors and vulnerabilities, such as elder abuse and social isolation. Mental illness in this age group is often exacerbated by diminished capacity and functional decline (21–23).

People with chronic illnesses, including heart disease (24,25), cancer (25–27), diabetes (25,28), and chronic pain (29,30), are more likely to experience mental health problems. The combination of physical and psychological distress can lead to social isolation, reduced quality of life, and exacerbation of both physical and mental health conditions (25,31). This can be exacerbate discomfort, increase feelings of fatigue and energy loss, and increase withdrawal from family, and non-adherence to medical management (24,32).

Individuals with physical disabilities are also at higher risk for mental health issues due to factors such as limited mobility, reduced social support, and environmental barriers (33,34). The study emphasizes the need for integrated healthcare services that address both physical and mental health, particularly for individuals with disabilities or chronic illnesses.

This study contributes valuable data on self-reported mental illness and its associated factors in Addis Ababa, providing a foundation for further research and interventions. However, the lack of standardized diagnostic tools is a limitation, and future studies should incorporate clinical assessments to confirm the prevalence and identify more specific mental health needs.

In conclusion, the study found a 1.1% prevalence of reported mental illness in Addis Ababa. Mental illness was more common among older adults, those with chronic illness, and individuals with physical disabilities. Despite the likelihood of underreporting, the findings highlight the significant burden of mental illness in the community. These results underscore the need for targeted mental health interventions and better integration of mental health services into healthcare, especially for vulnerable populations. Increasing community awareness and reducing stigma around mental illness are also essential.

## References

[1] World Health Organization (2024). Global burden of mental disorders and the need for a comprehensive, coordinated response from health and social sectors at the country level: report by the Secretariat.

[2] Atilola O, Singh Balhara YP, Stevanovic D, Avicenna M, Kandemir H (2013). Self-reported mental health problems among adolescents in developing countries: results from an international pilot sample. J Dev Behav Pediatr JDBP.

[3] Federal Democratic Republic of Ethiopia Ministry of Health (2012). National Mental Health Strategy (2012/13-2015/16). International Society of Substance Use Professionals.

[4] Habtamu Y, Admasu K, Tullu M, Kebede A (2022). Magnitude of common mental disorders and factors associated among people living in Addis Ababa Ethiopia 2018: community based cross-sectional study. BMC Psychiatry.

[5] Patel V, Flisher AJ, Hetrick S, McGorry P (2007). Mental health of young people: a global public-health challenge. Lancet Lond Engl.

[6] Huang Y, Loux T, Huang X, Feng X (2024). The relationship between chronic diseases and mental health: A cross-sectional study [Internet].

[7] Matandika I, Mategula D, Kasenda S, Adeniyi Y, Muula A (2024). Prevalence and correlates of common mental disorders among children and adolescents in Blantyre-Urban, Malawi.

[8] Terrill AL, Molton IR (2019). Frequency and impact of midlife stressors among men and women with physical disability. Disabil Rehabil.

[9] Hodgkinson S, Godoy L, Beers LS, Lewin A (2017). Improving Mental Health Access for Low-Income Children and Families in the Primary Care Setting. Pediatrics.

[10] Patel V, Kleinman A (2003). Poverty and common mental disorders in developing countries. Bull World Health Organ.

[11] Okoro CA, Strine TW, McKnight-Eily L, Verlenden J, Hollis ND Indicators of poor mental health and stressors during the COVID-19 pandemic, by disability status: A cross-sectional analysis.

[12] Arias D, Saxena S, Verguet S (2022). Quantifying the global burden of mental disorders and their economic value. EClinicalMedicine.

[13] Gruebner O, Rapp MA, Adli M, Kluge U, Galea S, Heinz A (2017). Cities and Mental Health. Dtsch Arzteblatt Int.

[14] Gureje O, Alem A (2000). Mental health policy development in Africa. Bull World Health Organ.

[15] Girma E, Ketema B, Mulatu T, Kohrt BA, Wahid SS, Heim E Mental health stigma and discrimination in Ethiopia: evidence synthesis to inform stigma reduction interventions. International Journal of Mental Health Systems.

[16] Engidaw NA, Abdu Z, Chinani I (2020). Prevalence and associated factors of common mental disorders among residents of Illu Ababore zone, southwest Ethiopia: a cross-sectional study. Int J Ment Health Syst.

[17] Birkie M, Anbesaw T (2021). Knowledge, attitude, and associated factors towards mental illness among residents of Dessie town, northeast, Ethiopia, a cross-sectional study. BMC Psychiatry.

[18] Jarso MH, Debele GR, Gezimu W, Nigatu D, Mohammedhussein M, Mamo A (2022). Knowledge, attitude, and its correlates of the community toward mental illness in Mattu, South West Ethiopia. Front Psychiatry.

[19] Gebrekidan Abbay A, Tibebe Mulatu A, Azadi H (2018). Community Knowledge, Perceived Beliefs and Associated Factors of Mental Distress: A Case Study from Northern Ethiopia. Int J Environ Res Public Health.

[20] Pilania M, Yadav V, Bairwa M, Behera P, Gupta SD, Khurana H (2019). Prevalence of depression among the elderly (60 years and above) population in India, 1997-2016: a systematic review and meta-analysis. BMC Public Health.

[21] World Health Organization (2024). Mental health [Internet].

[22] Wolde A, Wolancho W, Belay Y, Alemu A, Asefa A, Gebremedhin T (2022). A Community-Based Exploratory Cross-Sectional Study of Elder Abuse Perpetration or Victimization Among Elders in Ethiopia.

[23] Mm F, G C, Ss C, A E, S L, R L (2016). A systematic review of physical illness, functional disability, and suicidal behaviour among older adults. Aging Ment Health.

[24] De Hert M, Detraux J, Vancampfort D (2024). The intriguing relationship between coronary heart disease and mental disorders.

[25] Parrish E (2018). Comorbidity of mental illness and chronic physical illness: A diagnostic and treatment conundrum. Perspect Psychiatr Care.

[26] Shang X, Hodge AM, Peng W, He M, Zhang L (2020). Are Leading Risk Factors for Cancer and Mental Disorders Multimorbidity Shared by These Two Individual Conditions in Community-Dwelling Middle-Aged Adults?. Cancers.

[27] Massetti GM, Thomas CC, King J, Ragan K, Buchanan Lunsford N (2017). Mental Health Problems and Cancer Risk Factors Among Young Adults. Am J Prev Med.

[28] CDC (2024). Diabetes.

[29] D, Osterweis M, Kleinman A, Mechanic D (2024). Pain and Disability: Clinical, Behavioral, and Public Policy Perspectives.

[30] Psychiatry.org - Chronic Pain and Mental Health Often Interconnected (2024).

[31] Rosso AL, Taylor JA, Tabb LP, Michael YL (2024). Mobility, disability, and social engagement in older adults.

[32] Becerra BJ, Banta JE, Ghamsary M, Martin LR, Safdar N (2024). Burden of mental illness on hospital and patient outcomes among asthma hospitalizations.

[33] Hwang AW, Chang CH, Granlund M, Imms C, Chen CL, Kang LJ (2024). Longitudinal Trends of Participation in Relation to Mental Health in Children with and without Physical Difficulties.

[34] Marques Js, Regalado Icr, Galvão Érvp, Ferreira Hnc, Longo E, Lindquist Arr (2024). Participation in leisure activities from the perception of children with disabilities and their families in Brazil.

